# Withdrawn as Duplicate: The Impact of Concomitant Empiric Cefepime on Patient Outcomes of Methicillin-Resistant *Staphylococcus aureus* Bloodstream Infections Treated With Vancomycin

**DOI:** 10.1093/ofid/ofz077

**Published:** 2019-08-14

**Authors:** Evan J Zasowski, Trang D Trinh, Safana M Atwan, Marina Merzlyakova, Abdalhamid M Langf, Sahil Bhatia, Michael J Rybak

This manuscript has been withdrawn as a duplicate. The article is available at: https://doi.org/10.1093/ofid/ofz079 .

